# Braking and Propulsion Phase Characteristics of Traditional and Accentuated Eccentric Loaded Back Squats

**DOI:** 10.5114/jhk/185726

**Published:** 2024-04-15

**Authors:** Timothy J. Suchomel, Conor J. Cantwell, Brookelyn A. Campbell, Zachary S. Schroeder, Lauren K. Marshall, Christopher B. Taber

**Affiliations:** 1 Department of Human Movement Sciences, Carroll University, Waukesha, WI, USA.; 2 Directorate of Sport, Exercise, and Physiotherapy, University of Salford, Greater Manchester, UK.; 3 Department of Athletics, University of Wisconsin-Platteville, Platteville, WI, USA.; 4 Department of Athletics, University of Houston, Houston, TX, USA.; 5 Department of Athletics, Morningside University, Sioux City, IA, USA.; 6 Department of Fitness, Movement Fitness Rockford, Rockford, IL, USA.; 7 Department of Exercise Science, Sacred Heart University, Fairfield, CT, USA.

**Keywords:** strength, rate of force development, impulse, velocity-based training, power

## Abstract

The purpose of this study was to examine the differences in braking and propulsion force-time characteristics and barbell velocity between traditional (TRAD) and accentuated eccentric loaded (AEL) back squats using various load combinations. Sixteen resistance-trained men participated in four separate testing sessions which included a one repetition maximum (1RM) back squat during the first session and three squat testing sessions. During the squat testing sessions, participants either performed sets of three repetitions of TRAD back squats each with 50, 60, 70, and 80% 1RM or performed the same loads with the addition of weight releasers that increased the total eccentric weight of the first repetition of each set to either 100 (AEL-MAX) or 110% 1RM (AEL-SUPRA). Braking and propulsion mean force, duration, and impulse as well as mean and peak barbell velocity were compared between each condition and load. Significantly greater braking impulses were produced during the AEL-MAX and AEL-SUPRA conditions compared to TRAD (p < 0.03) with small-moderate effect sizes favoring AEL-SUPRA. No other significant differences existed among conditions for other braking, propulsion, or barbell velocity variables. AEL-MAX and AEL-SUPRA back squats may provide a greater braking stimulus compared to TRAD squats; however, the propulsion phase of the movement does not appear to be impacted. From a loading standpoint, larger and smaller load spreads may favor rapid and maximal force production characteristics, respectively. Further research on this topic is needed as a large portion of the braking stimulus experienced during AEL back squats may be influenced by relative strength.

## Introduction

While traditional (TRAD) resistance training exercises such as squatting, pressing, and pulling variations may serve as effective training stimuli for athletes, there has been a growing interest in the implementation of accentuated eccentric loading (AEL) within the last decade. AEL is characterized by the inclusion of heavier loads during the eccentric (lowering) phase compared to the concentric (upward) phase of an exercise, the pairing of eccentric and concentric muscle actions (i.e., stretch-shortening cycle), and minimal disruption of the natural movement mechanics of the exercise (Suchomel et al., 2019b; Wagle et al., 2017). The heavier loads during the eccentric phase of an exercise are typically provided by using weight releasers positioned on both sleeves of a barbell that contact the floor and drop off or by dropping weights (e.g., plates or dumbbells) at the bottom of a countermovement prior to a jump. Through cross-sectional studies, researchers have shown that AEL may provide an effective eccentric stimulus compared to TRAD loading during the back squat and bench press exercises (Lates et al., 2022; Wagle et al., 2018, 2021), but may also provide a within-set potentiation effect leading to an enhanced concentric performance (Lates et al., 2022; Merrigan et al., 2020; Taber et al., 2021). Given the potential of AEL stimuli, it should come as no surprise that training studies lasting 5–10 weeks led to improvements in maximal strength (Brandenburg and Docherty, 2002; Douglas et al., 2018; Munger et al., 2022; Walker et al., 2016; Yarrow et al., 2008) and power output (Sheppard et al., 2008).

Despite the existing literature favoring the use of AEL, there is limited information that discusses best practices for implementing this training method. Although a recent review summarized the existing AEL literature to provide general training recommendations (Merrigan et al., 2022), further information focused on the eccentric and concentric loading of an exercise using AEL is needed. Aside from ballistic jumping exercises that typically use much lighter training loads (Bridgeman et al., 2017; Sheppard et al., 2007; Taber et al., 2023), researchers have examined various eccentric/concentric load combinations with squatting variations including 105/80% of the participants’ one repetition maximum (1RM) during back squats (Wagle et al., 2018, 2021) and 120/90% 1RM during front squats (Munger et al., 2017) as well as 105/80% 1RM (Lates et al., 2022), 120/80% and 120/65% 1RM (Merrigan et al., 2020), and 120/100% 1RM during the bench press (Ojasto and Häkkinen, 2009). It should be noted that while one group of researchers indicated the eccentric and concentric loading may have an impact on within-set potentiation effects (Merrigan et al., 2020), none of the other groups listed above examined a spectrum of loading combinations. However, Taber and colleagues (2021) investigated eccentric loads of 100 and 110% 1RM and concentric loads ranging from 30 to 80% 1RM in 10% increments during the bench press exercise. The authors concluded that eccentric loads of 100 and 110% 1RM could both enhance the mean barbell velocity of the concentric phase, but the effects might be contingent on both the eccentric load and the spread between eccentric and concentric loads. Although these findings are important, limited information exists on how various loading combinations impact squat performance. Moreover, no information currently exists on how the force production characteristics of an exercise may be affected by different load combinations.

Since squatting variations are frequently implemented in resistance training programs (Comfort et al., 2018), and because previous findings support the use of AEL for improving strength-power characteristics, further research is needed on how different load combinations impact the overall training stimulus provided. Therefore, the purpose of this study was to examine the differences in braking and propulsion force-time characteristics and barbell velocity between TRAD and AEL squats using different load combinations. It was hypothesized that AEL squats using weight releasers would provide a superior eccentric stimulus and enhance the performance of subsequent repetitions compared to TRAD squats performed with the same concentric loads.

## Methods

### Design

**Figure 1:**
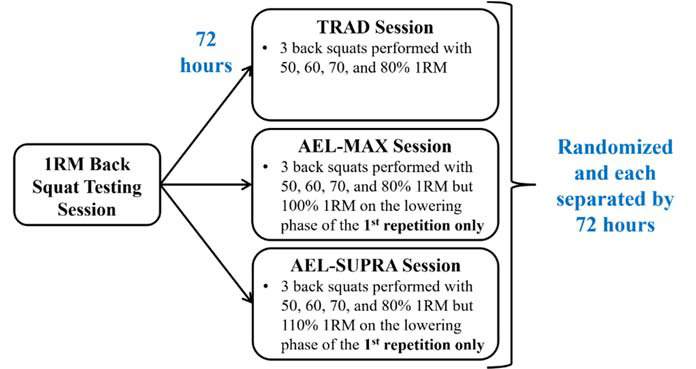
Testing order sequence.

A randomized, repeated measures design was used to examine the differences in the force-time and barbell velocity characteristics between TRAD back squats and those performed using maximal (AEL-MAX; 100% 1RM) or supramaximal (AEL-SUPRA; 110% 1RM) AEL loading during the first repetition of a set. Each participant completed four separate testing sessions over the course of 10 days and individual testing sessions were held at approximately the same time of the day (within a two-hour time range) to account for changes in circadian rhythms (Figure 1). Braking and propulsion mean force, duration, and impulse, as well as mean (MBV) and peak barbell velocity (PBV), were measured using force plate and linear position transducer technology, respectively, and compared during the back squat sets performed with propulsion (concentric) loads of 50, 60, 70, and 80% 1RM.

### Participants

Sixteen resistance-trained men (age = 24.4 ± 3.8 years, body mass = 85.8 ± 12.3 kg, body height = 178.1 ± 7.9 cm, 1RM back squat = 168.0 ± 23.7 kg, relative 1RM back squat = 2.0 ± 0.3 kg/kg) participated in the experiment. Prior to this study, each participant consistently trained at least three times per week while incorporating the back squat exercise into their training sessions for a minimum of one year. This study was approved by the Institutional Review Board of the Carroll University (protocol code: #21-044, approval date: 30 November 2021) and each participant provided written informed consent before participating in any testing session. All procedures were in accordance with the Declaration of Helsinki.

### Procedures

After arriving at the laboratory, each participant completed the informed consent procedures before their age, body mass, height, and estimated 1RM back squat data were collected. Participants then performed a standardized warm-up that included three minutes of light-moderate stationary cycling and dynamic stretching (Suchomel et al., 2019a, 2023). After the general warm-up, participants began a specific back squat warm-up protocol that consisted of self-selected repetitions with an empty barbell and five repetitions at 30%, five repetitions at 50%, three repetitions at 70%, and one repetition at 90% of their estimated 1RM (Suchomel et al., 2016). Following the warm-up protocol, the loads for the 1RM attempts were adjusted by the principal investigator and research assistants using a minimum increase of 2.5 kg. Participants were provided with two minutes of rest between the first two warm-up sets while 3–5 min were provided following the final two warm-up sets and between the 1RM attempts. An acceptable 1RM attempt required participants to squat to a depth where the top of their thighs, at a minimum, were parallel to the floor. Squat depth was visually monitored by the principal investigator and research assistants, and participants achieved their 1RM within five maximal attempts or less.

Following the 1RM test, participants were provided with a self-selected rest interval before completing familiarization back squat trials with weight releasers (Monster Grips, Columbus, OH). First, participants performed back squat repetitions with an empty 20-kg barbell and weight releasers (5 kg each) positioned on each sleeve of the barbell. The principal investigator and research assistants adjusted the weight releaser height to allow them to contact the ground and fall off the barbell just above the participant’s full squat position. This allowed participants to experience the added load of the weight releasers through the largest range of motion while also allowing the weight releasers to be safely removed with minimal disruption to the participant’s movement mechanics. After performing empty barbell repetitions, participants performed a set of three repetitions with 50% of their 1RM back squat on the barbell with the addition of empty weight releasers on the first repetition. Finally, participants performed three back squat repetitions with 50% 1RM on the barbell and weight releaser weight that represented 10% 1RM on each side (i.e., 70% 1RM total). Using this set up, the eccentric phase of the first repetition of the set was performed with 70% 1RM, while the propulsion phase was performed with 50% 1RM and the eccentric/concentric phase of the subsequent two repetitions was performed with 50% 1RM.

Participants returned for their first of three squat testing sessions 72 h following their 1RM testing and familiarization session. The sessions included either TRAD, AEL-MAX, or AEL-SUPRA squats. Each session was separated by 72 h and the order of the testing sessions was randomized to prevent an order effect. Briefly, upon arrival, the body mass information of participants was collected before they completed the standardized warm-up described above. Following the warm-up, participants performed a self-selected number of repetitions with an empty 20-kg barbell. During the AEL sessions, empty weight releasers were added to the barbell after the warm-up repetitions and the participant performed a set of three repetitions with the weight releasers falling off on the first repetition to provide a final familiarization trial and to confirm the weight releaser length. Following the empty barbell repetitions, participants performed a back squat warm-up protocol that included five repetitions with 30%, three repetitions with 50%, and three repetitions with 70% of their 1RM with a two-minute rest interval between each set. During AEL sessions, it is important to note that participants also performed a “walk-out” with the first testing load on the barbell (i.e., 50% 1RM) and the additional weight releaser load. This was done for safety purposes and to allow participants to become familiar with the eccentric load and the potential swing of the weight releasers. After the warm-up was completed, each participant performed a set of three repetitions with 50, 60, 70, and 80% 1RM in a progressive order with a three-minute rest interval between each set. It should be noted that the weight releasers were only used on the first repetition of each set during the AEL conditions. Prior to the testing repetitions, participants were instructed to perform the eccentric phase of each repetition using their natural squatting tempo and the concentric phase as fast as possible. During each trial, participants would unrack the barbell, step onto the force plate, and stand motionless for at least one second. After receiving a countdown of “3, 2, 1, Go!”, participants would perform three maximal effort back squat repetitions before re-racking the barbell. Strong verbal encouragement was provided during each repetition to ensure maximal effort.

During the TRAD session, the eccentric and concentric phases of each repetition were performed with the same load. In contrast, the eccentric phase of the first repetition of each back squat set was performed with the equivalent of 100 or 110% during the AEL-MAX or AEL-SUPRA sessions, respectively; however, the concentric load of each repetition was the same as the TRAD session (Wagle et al., 2018, 2021). Thus, within the AEL-MAX and AEL-SUPRA sessions, different percentages of the participant’s 1RM were loaded onto the weight releasers and fell off at the end of the eccentric phase of the first repetition. For example, during the AEL-MAX session, the participant would perform the eccentric phase with 50% 1RM on the barbell and 50% 1RM distributed between the weight releasers (i.e., 25% 1RM each) during the first repetition of the set before the weight releasers were removed. In this scenario, the participant would then perform the concentric phase of the first repetition and the remaining two repetitions with 50% 1RM. As the barbell load increased during the following sets, a smaller percentage of the total load was removed.

### Data Analyses

**Figure 2:**
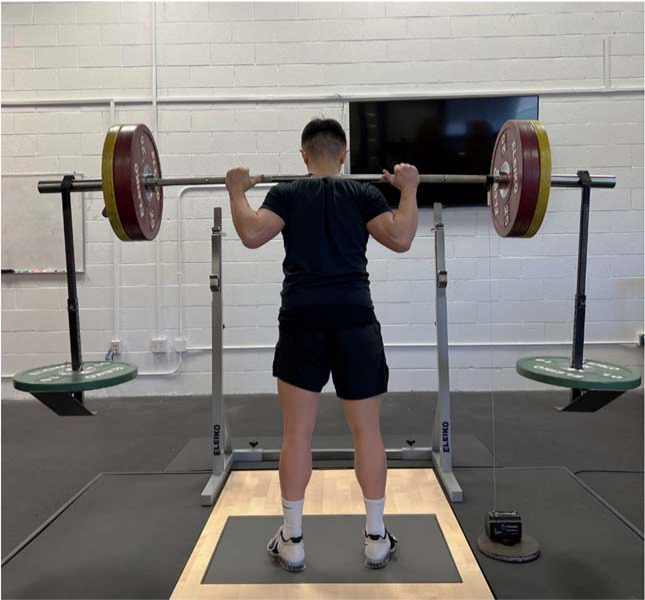
Force plate and linear position transducer set up during a back squat performed with accentuated eccentric loading.

**Figure 3:**
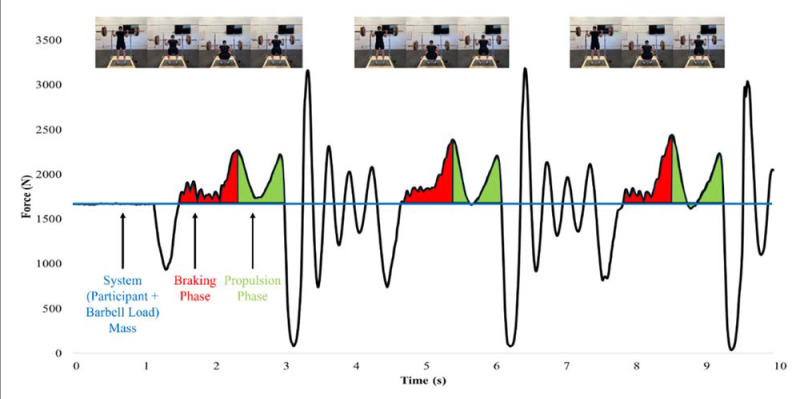
Force-time curve example during a traditional back squat set.

**Figure 4:**
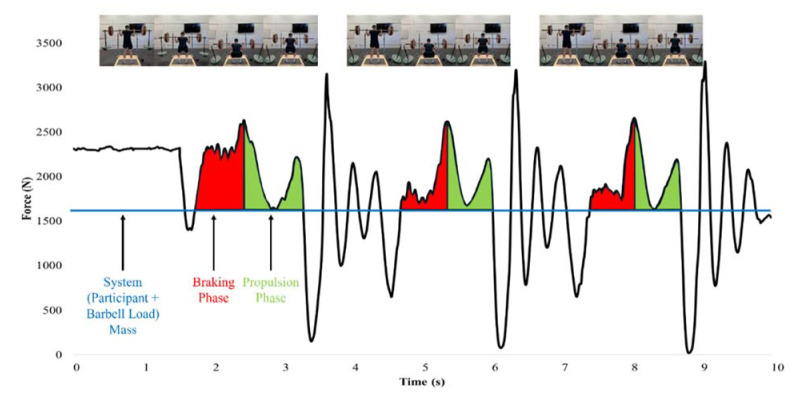
Force-time curve example during a back squat set performed with accentuated eccentric loading.

Each back squat repetition was performed on a force plate (Model 6090-06, Bertec Corporation, Columbus, OH, USA) sampling at 1000 Hz with a linear position transducer (GymAware Powertool, Kinetic Performance Technology, Braddon, Australia) attached to the barbell that used a variable sampling rate with level crossing detection (Figure 2). The raw force-time data were exported to a custom spreadsheet (Microsoft Inc., Redmond, WA, USA) to determine the net mean force, duration, and net impulse of the braking and propulsion phases of each back squat repetition. The start of the braking phase was identified as the point at which force exceeded system mass (participant’s body mass + concentric barbell load) following the unweighting phase, whereas the end of the braking phase was identified as the lowest position of the squat (identified by the linear position transducer) and where the greatest braking force occurred. The start of the propulsion phase was identified as the point following the peak braking force, while the end was identified as the last force value produced above the system mass. Examples of TRAD and AEL squat force-time curve analyses are displayed in Figures 3 and 4. Net mean force was calculated as the average force produced during the braking and propulsion phases, while phase duration was determined as the length of time of each phase. Finally, braking and propulsion net impulses were calculated as the products of net mean force and duration of the respective phases. Relative mean force was calculated by dividing net mean force by the body mass of the participant. The average braking and propulsion mean force, duration, and impulse produced across all three squat repetitions was used for statistical analysis. MBV and PBV data were measured by the linear position transducer which was connected via Bluetooth to a tablet (iPad 2, Apple Inc., Cupertino, CA, USA) using the most recent GymAware application version. Velocity-time data were calculated by dividing the measured displacement by the movement time. The MBV and PBV of each squat repetition were determined as the average and peak values calculated during the propulsion phase of the movement, respectively. The average MBV and PBV values produced across each set were used for statistical comparison.

### Statistical Analyses

The distribution of the dependent variable data was assessed using the Shapiro-Wilk normality test. Relative test-retest reliability of the data was examined using two-way, mixed intraclass correlation coefficients (ICC). It should be noted that the test-retest reliability of braking and propulsion mean force, duration, and impulse was assessed across all the three repetitions of the TRAD squats as the influence of AEL may modify the performance of subsequent repetitions (Wagle et al., 2018). Poor, moderate, good, and excellent ICC data coincided with lower bound 95% confidence intervals of <0.50, 0.50–0.74, 0.75–0.90, and >0.90, respectively (Koo and Li, 2016). A series of 3 (condition) x 4 (load) repeated measures ANOVA with Bonferroni post hoc comparisons were used to compare the braking and propulsion force-time and barbell velocity characteristics produced during the TRAD and AEL back squats performed with concentric loads of 50, 60, 70, and 80% 1RM. If the assumption of sphericity was violated, Greenhouse-Geisser adjusted values were reported. Hedge’s g effect sizes were calculated to examine the magnitude of the differences among conditions and loads. Effect sizes were considered trivial, small, moderate, large, very large, and nearly perfect when the values were 0.00–0.19, 0.20–0.59, 0.60–1.19, 1.20–1.99, 2.00–3.99, and ≥ 4.00, respectively (Hopkins, 2014). A criterion alpha of *p* ≤ 0.05 was used to establish statistical significance and all statistical tests were completed using SPSS 28 (IBM, Chicago, IL, USA).

## Results

All the braking and propulsion force plate and barbell velocity data were normally distributed and are displayed in Tables 1–3. The ICC data ranged from 0.82 to 0.98 and 0.75 to 0.97 for force plate and barbell velocity data, respectively.

**Table 1:** Descriptive braking data for traditional (TRAD), maximal accentuated eccentric loading (AEL-MAX), and supramaximal AEL (AEL-SUPRA) back squats. % 1RM based on the concentric load used during the movement; BMF = net braking mean force; BDur = braking duration; BImp = net braking impulse; * = significantly greater than TRAD (*p* < 0.03); a = significantly different from values at 80% 1RM (*p* < 0.001); b = significantly different from values at 70% 1RM (*p* < 0.001); c = significantly different from values at 60% 1RM (*p* < 0.001)

Condition	BMF (N•kg^−1^)	BDur (s)	BImp (Ns)
50% 1RM^a,b,c^			
TRAD	5.4 ± 1.7	0.50 ± 0.12	210.7 ± 27.3
AEL-MAX	6.5 ± 1.1	0.55 ± 0.08	304.6 ± 24.6*
AEL-SUPRA	6.9 ± 1.2	0.55 ± 0.10	328.1 ± 66.8*
60% 1RM^a,b^			
TRAD	5.0 ± 1.5	0.55 ± 0.11	216.8 ± 29.0
AEL-MAX	6.2 ± 1.0	0.55 ± 0.08	284.9 ± 41.9*
AEL-SUPRA	6.4 ± 1.0	0.57 ± 0.08	316.5 ± 42.6*
70% 1RM^a^			
TRAD	4.4 ± 1.1	0.61 ± 0.11	215.6 ± 30.6
AEL-MAX	5.5 ± 0.8	0.61 ± 0.09	280.7 ± 43.1*
AEL-SUPRA	5.7 ± 0.7	0.63 ± 0.08	311.8 ± 48.0*
80% 1RM			
TRAD	3.8 ± 0.9	0.71 ± 0.11	222.2 ± 37.7
AEL-MAX	4.6 ± 0.7	0.68 ± 0.08	260.5 ± 37.5*
AEL-SUPRA	5.0 ± 0.8	0.69 ± 0.10	289.6 ± 41.0*

**Table 2:** Descriptive propulsion data for traditional (TRAD), maximal accentuated eccentric loading (AEL-MAX), and supramaximal AEL (AEL-SUPRA) back squats. % 1RM based on the concentric load used during the movement; PMF = net propulsion mean force; PDur = propulsion duration; PImp = net propulsion impulse; a = significantly different from values at 80% 1RM (*p* < 0.001); b = significantly different from values at 70% 1RM (*p* < 0.001); c = significantly different from values at 60% 1RM (*p* < 0.001)

Condition	PMF (N•kg^−1^)	PDur (s)	PImp (Ns)
50% 1RM^a,b,c^			
TRAD	4.9 ± 0.9	0.57 ± 0.08	229.6 ± 29.5
AEL-MAX	4.8 ± 1.0	0.60 ± 0.08	236.7 ± 31.7
AEL-SUPRA	4.9 ± 0.8	0.58 ± 0.10	234.3 ± 36.7
60% 1RM^a,b^			
TRAD	4.2 ± 0.8	0.67 ± 0.09	244.7 ± 31.8
AEL-MAX	4.2 ± 0.8	0.73 ± 0.12	253.1 ± 26.4
AEL-SUPRA	4.3 ± 0.8	0.70 ± 0.11	248.3 ± 29.7
70% 1RM^a^			
TRAD	3.6 ± 0.8	0.86 ± 0.13	250.3 ± 38.2
AEL-MAX	3.6 ± 0.8	0.87 ± 0.16	252.5 ± 28.6
AEL-SUPRA	3.6 ± 0.6	0.87 ± 0.16	257.9 ± 30.6
80% 1RM			
TRAD	2.8 ± 0.7	1.14 ± 0.21	261.0 ± 32.6
AEL-MAX	2.8 ± 0.6	1.20 ± 0.26	271.1 ± 36.4
AEL-SUPRA	2.9 ± 0.7	1.17 ± 0.28	274.3 ± 35.0

**Table 3:** Descriptive barbell velocity data for traditional (TRAD), maximal accentuated eccentric loading (AEL-MAX), and supramaximal AEL (AEL-SUPRA) back squats. % 1RM based on the concentric load used during the movement; MBV = mean barbell velocity; PBV = peak barbell velocity; a = significantly different from values at 80% 1RM (*p* < 0.001); b = significantly different from values at 70% 1RM (*p* < 0.001); c = significantly different from values at 60% 1RM (*p* < 0.001)

Condition	MBV (m•s^−1^)	PBV (m•s^−1^)
50% 1RM^a,b,c^		
TRAD	0.88 ± 0.08	1.42 ± 0.18
AEL-MAX	0.86 ± 0.08	1.37 ± 0.17
AEL-SUPRA	0.86 ± 0.08	1.36 ± 0.19
60% 1RM^a,b^		
TRAD	0.77 ± 0.07	1.28 ± 0.17
AEL-MAX	0.76 ± 0.06	1.25 ± 0.15
AEL-SUPRA	0.77 ± 0.07	1.28 ± 0.17
70% 1RM^a^		
TRAD	0.64 ± 0.08	1.14 ± 0.17
AEL-MAX	0.65 ± 0.07	1.13 ± 0.12
AEL-SUPRA	0.66 ± 0.07	1.16 ± 0.16
80% 1RM		
TRAD	0.51 ± 0.06	1.02 ± 0.13
AEL-MAX	0.50 ± 0.07	1.03 ± 0.14
AEL-SUPRA	0.52 ± 0.08	1.04 ± 0.15

### Interaction Effects

There were significant condition x load interaction effects found for braking duration and impulse (both *p* < 0.001) as well as PBV (*p* = 0.013); however, there were no significant interaction effects for braking mean force (*p* = 0.395), propulsion mean force (*p* = 0.831), duration (*p* = 0.413), or impulse (*p* = 0.628). Furthermore, there were no significant condition x load interaction effects for MBV (*p* = 0.404). Post hoc comparisons are displayed in Table 1. There were trivial-small effects among all squat conditions for braking duration (g = 0.01–0.57). In contrast, large (g = 1.67–2.24) and moderate-large effects (g = 0.99–3.52) were present when comparing the braking impulse between AEL-SUPRA and AEL-MAX with TRAD, respectively. In addition, small-moderate effects (g = 0.45–0.73) favored the AEL-SUPRA condition when compared to AEL-MAX for braking impulse. Despite a lack of statistical significance, there were large (g = 1.67–2.24) and moderate (g = 0.77–1.16) effects that favored AEL-SUPRA and AEL-MAX for braking mean force compared to TRAD, respectively; however, only small effects were present when comparing AEL conditions (g = 0.25–0.51). For propulsion variables, only trivial-small effects were present among all conditions for mean force (g = 0.01–0.17), duration (g = 0.02–0.43), and impulse (g = 0.07–0.38). Similarly, trivial-small effects existed among all conditions for MBV (g = 0.02–0.27) and PBV (g = 0.02–0.32).

### Main Effects

There were significant condition main effects for braking mean force (*p* < 0.001), while there were no differences when examining braking duration (*p* = 0.551) or propulsion mean force (*p* = 0.844), duration (*p* = 0.168), or impulse (*p* = 0.356). In addition, there were no significant condition main effect differences for MBV (*p* = 0.542) or PBV (*p* = 0.543). The pairwise comparison analysis showed that braking mean forces produced during AEL-SUPRA were significantly greater than those during both TRAD (*p* < 0.001) and AEL-MAX (*p* = 0.017). In addition, greater braking mean forces were produced during AEL- MAX compared to TRAD (*p* = 0.001). Finally, significant load main effects were present for all the examined variables (all *p* < 0.001). Post hoc comparisons are shown in Tables 1–3.

## Discussion

The current study examined the differences in braking and propulsion force-time characteristics and barbell velocities between TRAD and AEL back squat sets performed with a spectrum of relative barbell loads. The primary finding of this study was that significantly greater braking impulses were produced during the AEL-MAX and AEL-SUPRA squat conditions compared to TRAD across every loading condition. These findings appeared to be underpinned by a significant condition main effect which showed that greater braking mean forces were produced during the AEL conditions. In contrast, there were no significant differences between conditions for braking duration nor any propulsion force-time or barbell velocity variable. Finally, and as expected, there was a significant load main effect for every variable indicating that the load had a meaningful impact on braking and propulsion force, duration, and impulse as well as barbell velocity.

The greatest braking stimulus was provided by the AEL-SUPRA condition as evidenced by the greatest braking mean forces and impulses produced over similar durations as the other conditions. Specifically, the differences in braking mean forces and impulses produced during the AEL-SUPRA condition compared to TRAD were moderate-large and large across the loads examined, respectively. Similar results were displayed by researchers who used 105% 1RM during the eccentric phase and 80% 1RM during the concentric phase of the back squat exercise (Wagle et al., 2018, 2021). Interestingly, participants also produced practically meaningful differences regarding braking mean force and impulse during the AEL-MAX condition compared to TRAD; however, it should be noted that small and small-moderate effects for braking mean force and impulse favored the AEL-SUPRA compared to the AEL-MAX condition, respectively. Based on the current results, AEL may allow participants to develop their braking characteristics to a greater extent than TRAD loading by producing greater forces over similar movement durations, thus creating a larger “peaked” impulse (i.e., greater force and rate of force development). Moreover, it also appears that back squats may not always need to use AEL-SUPRA loading during the eccentric phase of the lift to provide an enhanced braking stimulus; however, it may be beneficial to athletes that can tolerate and actively resist such loading.

In contrast to our hypothesis, both the AEL-MAX and AEL-SUPRA conditions failed to enhance the propulsion phase of the back squat repetitions across the loads examined. In fact, only trivial-small effects existed between the TRAD and AEL conditions for propulsion mean force, duration, and impulse as well as MBV and PBV. The current findings are in line with previous research on the back squat (Wagle et al., 2018, 2021), but contradict additional research on the bench press (Lates et al., 2022; Merrigan et al., 2020; Taber et al., 2021). A possible explanation for a lack of within-set potentiation may be due to the comparisons made between the average performance across all three repetitions within each set compared to an individual repetition analysis. Wagle et al. (2018) indicated that AEL used only during the first repetition of a five repetition set might enhance the eccentric rate of force development for only the first three repetitions. Thus, given the unique AEL stimuli examined in this study compared to others, additional analyses may be warranted to determine whether individual repetitions were impacted.

Another potential explanation for the lack of significant propulsion variable findings may be due to the wide range of back squat relative strength within the sample of participants (1.44–2.47 times body weight). Upon examining the relationships between relative strength and the braking impulse generated across the AEL sets, a large portion of the variance was explained. Specifically, 46.4–67.1% and 45.6–62.1% of the braking impulse variance was explained by relative strength during the AEL-MAX and AEL-SUPRA sets, respectively. Based on these findings and the conclusions of additional literature (Suchomel et al., 2018, 2019c), AEL as a training method, especially AEL-MAX and AEL-SUPRA, should be considered an advanced training strategy. Merrigan and colleagues (2021) support this notion as the authors indicated that the within-set potentiation effects displayed with AEL bench press sets may be contingent on the relative strength of participants. Conclusions from potentiation literature that used heavy squatting variations further support this idea (Seitz et al., 2014; Suchomel et al., 2016). Despite the current literature, there is a lack of information regarding the force-time characteristics produced by stronger and weaker participants during AEL exercise. Therefore, it is recommended that researchers consider investigating these differences.

To the authors’ knowledge, this is the first study to examine the braking and propulsion force-time characteristics of back squats performed with a spectrum of relative loads and various load combinations with AEL-MAX and AEL-SUPRA. Large braking net mean forces were produced during shorter durations when the load spread (i.e., difference between the AEL total load and the barbell load) was larger during each AEL condition. These results suggest that larger load spreads during AEL back squats may benefit the braking rate of force development characteristics of athletes. Moreover, if individuals using AEL back squats have the capacity to tolerate greater rapid force production during the braking phase, it is possible that these characteristics may translate to an enhanced propulsion rate of force development characteristics as indicated by large net mean forces and shorter durations with lighter concentric loads. Regarding the development of maximal force production and strength characteristics, smaller load spreads during AEL squats may be favorable due to the larger absolute loads experienced throughout the movement. While researchers examined eccentric loads of 100 and 110% 1RM combined with 30–80% 1RM concentric loads during the bench press (Taber et al., 2021), the current results cannot be directly compared and thus, it is suggested that further research on the loads and load spreads used during AEL is needed.

The current study is not without its limitations. First, only resistance-trained men were included within the analyses; thus, the findings of this study can only be generalized to related populations. To the authors’ knowledge, minimal research has examined the use of AEL with female participants (Harden et al., 2020; Sheppard et al., 2008); however, further research is needed to improve the prescription of AEL for this population. Second, only one set of AEL-MAX and AEL-SUPRA was performed using each load combination. While investigating AEL in this manner may provide evidence to support the use of various load combinations, it is currently unknown whether the current results can be replicated over the course of multiple sets. Thus, it is recommended that researchers examine different AEL load combinations performed over several sets to simulate a resistance training session. Finally, in contrast to previous research (Wagle et al., 2018, 2021), the current study only used AEL during the first repetition of each set. Therefore, additional research is needed to determine whether performing AEL on multiple repetitions may provide a greater training stimulus using the spectrum of load combinations applied in this study.

The findings of the current study support the notion that AEL squats using maximal (i.e., 100% 1RM) and supramaximal (i.e., 110% 1RM) loading during the eccentric phase of the movement may provide a greater braking stimulus compared to TRAD back squats due to greater mean forces and impulses being produced. However, the AEL-MAX and AEL-SUPRA back squats did not appear to enhance the propulsion phase of the movement regardless of the concentric load on the barbell (i.e., 50–80% 1RM) compared to TRAD squats. Larger load spreads may provide an effective rapid force production stimulus, whereas smaller load spreads may benefit maximal force and strength characteristics to a greater extent than the alternative. The relative strength of an athlete may explain a significant portion of the braking stimulus experienced during AEL back squats, which provides further evidence that AEL may be considered an advanced training method.
